# Association between alcohol consumption and allergic rhinitis in US adults

**DOI:** 10.3389/fnut.2025.1544889

**Published:** 2025-03-21

**Authors:** Yan He, Yingying Xu, Zhiqiang Lin

**Affiliations:** ^1^Department of Otolaryngology Head and Neck Surgery, Affiliated Suzhou Hospital of Nanjing Medical University, Suzhou, China; ^2^Gusu School Nanjing Medical University, Suzhou, China; ^3^Department of Otolaryngology Head and Neck Surgery, The Second Affiliated Hospital of Jiangxi Medical College, Nanchang University, Nanchang, China

**Keywords:** allergic rhinitis, allergy, alcohol, alcohol consumption, NHANES

## Abstract

**Introduction:**

Increasing evidence suggests that alcohol consumption may be associated with allergic diseases. This cross-sectional analysis aimed to determine the correlation between alcohol consumption patterns and allergic rhinitis (AR) in US adults.

**Methods:**

A cross-sectional study was conducted involving 2,179 individuals aged 20 years and older who took part in the 2005–2006 National Health and Nutrition Examination Survey (NHANES), which assessed AR and alcohol consumption patterns. Alcohol consumption was categorized into three groups: “Never” (fewer than 12 drinks in a lifetime), “Now” (currently drinking), and “Former” (a prior history of drinking but no longer consuming alcohol). The association between alcohol consumption patterns and AR was analyzed separately for men and women, adjusting for several comorbidities.

**Results:**

Individuals who currently consume alcohol are more likely to exhibit elevated levels of total IgE and cat/dog dander-specific IgE compared to non-consumers. Compared to “Never” in the male group, “Now” (currently drinking) was positively associated with AR in both the partially adjusted analysis and the fully adjusted model. However, we did not find any positive relationship between alcohol consumption patterns and AR in the female group, which suggests that current drinking was linked to a higher prevalence of AR in men but not in women.

**Conclusion:**

We discovered that current drinking was positively associated with a high prevalence of AR in men.

## 1 Introduction

Allergic rhinitis (AR) is characterized by clinical symptoms of the upper airway, including paroxysmal sneezing, nasal pruritus, nasal obstruction, and rhinorrhea (1). These symptoms result in sleep disturbances, fatigue, depression, reduced olfactory perception, and cognitive impairment, all of which significantly affect the quality of life and productivity in both learning and work settings (2). Furthermore, AR is associated with the presence of asthma, eczema, chronic or recurrent sinusitis, coughing, and both tension and migraine headaches (3). Currently, AR affects approximately 15% of the US population (3) and costs the healthcare system $US4.9 billion annually (4). The increasing incidence of AR and other allergic diseases is attributed to lifestyle factors and environmental changes that influence immune regulation, increase susceptibility to allergy sensitization, and promote chronic inflammation (5).

As a global public health phenomenon, alcohol consumption is the third leading cause of premature mortality in the US (6). Alcohol-induced injury represents a systemic insult associated with both acute and chronic alcohol consumption, which contributes to increased comorbidities across various organ systems, including the nervous, respiratory, and digestive systems (7). Epidemiological studies indicate that alcohol may be a cofactor affecting allergic reactions (8). Previous research suggested that alcohol consumption is linked to a higher risk of developing AR and positively affects serum total immunoglobulin E (IgE) levels (9–11), which play a critical role in allergic diseases (12). Additionally, alcohol consumption during pregnancy may increase the incidence of early-onset atopic dermatitis in predisposed infants (13). However, according to a Mendelian randomization approach, there is no evidence that prenatal alcohol exposure raises the incidence of childhood asthma or atopy (14). Meanwhile, a prospective investigation discovered that, in contrast to exercise and aspirin, which enhance clinical responses in wheat-dependent exercise-induced anaphylaxis by decreasing the threshold and intensifying the allergic reaction, alcohol yielded ambiguous results (15). Inconsistent findings from earlier studies suggest that the relationship between alcohol consumption and allergic diseases remains unclear.

The National Health and Nutrition Examination Survey (NHANES) is a publicly available database. This study aimed to investigate whether alcohol consumption is related to AR in a sizable representative US adult sample.

## 2 Methods

### 2.1 Database and study subjects

The data analyzed were obtained from the NHANES database, which collects information on the diet, nutritional status, health, and health-related behaviors of the non-institutionalized US civilian population to assess health and nutrition. Before participating in the study, each subject provided written informed consent. They were first interviewed at home and then scheduled for examinations at the mobile examination center (MEC).

**Figure 1:**
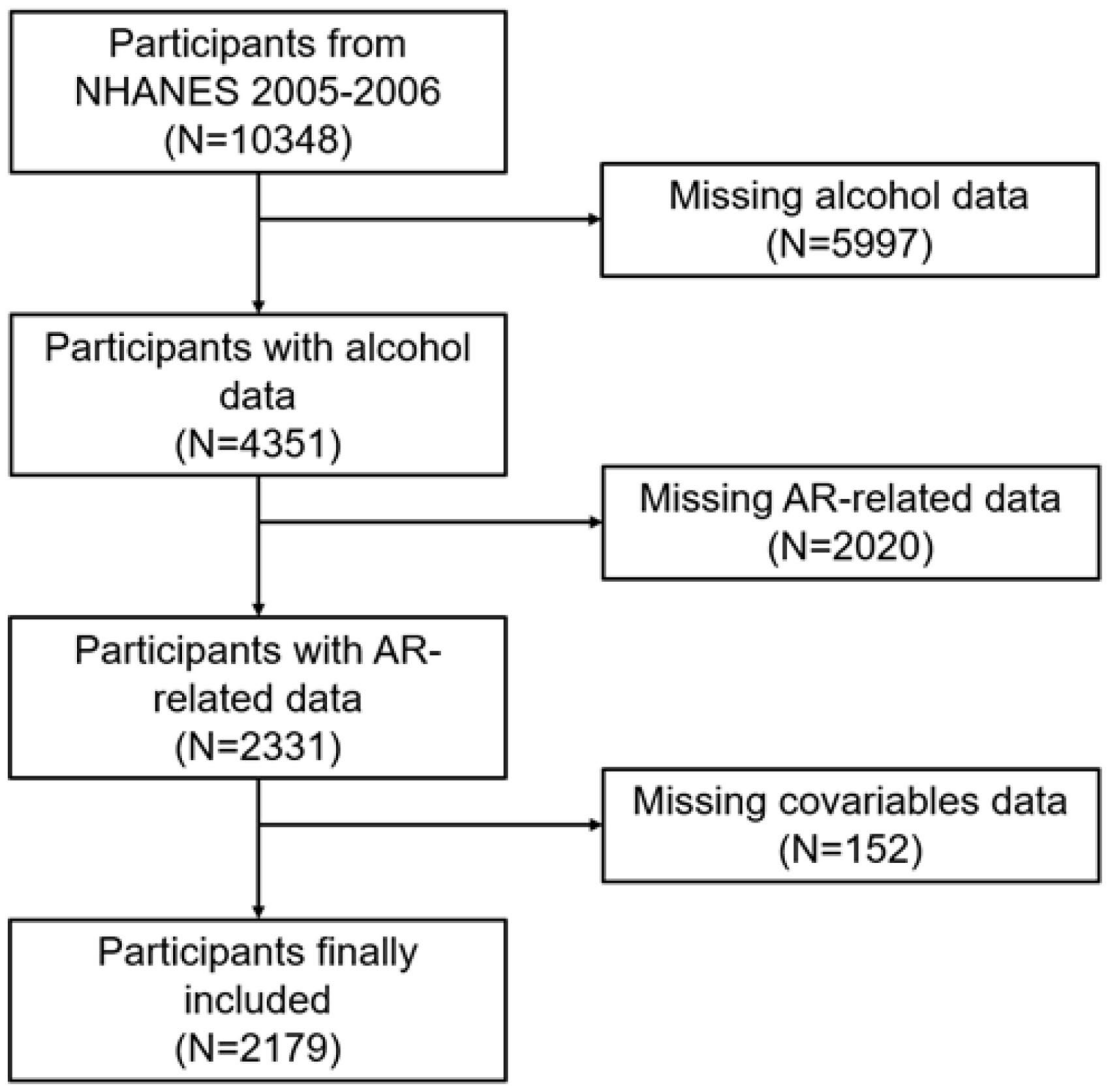
Sample selection process.

In this research, publicly accessible data from the 2005–2006 NHANES cycle were used. Initially, a total of 10,348 subjects were included. Subsequently, we excluded participants with missing data on alcohol consumption patterns (*n* = 5,997), those without AR-related data (*n* = 2,020), and those without covariate data (*n* = 152). Finally, 2,179 participants were included in our analysis (Figure 1).

### 2.2 Alcohol use

The questionnaire on alcohol use is administered to individuals aged 20 years and older. An alcoholic beverage is defined as a 12-ounce beer, a 5-ounce glass of wine, or 1.5 ounces of spirits.

Alcohol consumption patterns were the exposure variable in our study. Its definition was based on the following questionnaires: “ALQ101 - Had at least 12 alcoholic drinks in 1 year?” and “ALQ110 - Had at least 12 alcoholic drinks in a lifetime?” Participants were categorized as follows: “Never” (less than 12 alcoholic drinks in a lifetime) was defined as “no” to ALQ110; “Now” (currently drinking) was defined as “yes” to ALQ101; and “Former” (prior history of drinking but no longer drinking) was defined as “yes” to ALQ110 but “no” to ALQ101.

In addition, ALQ130 (average alcoholic drinks/day in the past 12 months) was used to assess the average alcohol intake. “Heavy drinking” was defined as responding “yes” to ALQ050 (Ever have five or more drinks every day?).

### 2.3 Assessment of AR

AR diagnosis was based on a combination of questionnaires and 19 allergen-specific IgE levels. The 2005–2006 NHANES survey included inquiries related to prevalent allergic diseases. The AR symptom was classified as “yes” in response to either of the following two inquiries: “AGQ030: During the past 12 months, have you had an episode of hay fever?” and/or “AGQ100: During the past 12 months, have you had a problem with sneezing, or a runny or blocked nose when you did not have a cold or the flu?” A total of 19 allergen-specific IgE levels were measured, with the lower limit of detection remaining constant at 0.35 kU/L. The inclusion criteria for AR participants included a positive serum IgE history and a “yes” response to either of the two rhinitis-related questions. In contrast, the inclusion criteria for the control group were participants with a negative serum IgE history and with “yes” responses to neither of the two rhinitis-related questions. The other participants were excluded from the analyses.

### 2.4 Covariates of interest

To assess the possible influence of variables that may affect the outcomes, relevant factors were incorporated into our multivariable-adjusted models, informed by prior research (6, 16, 17). The variables considered as covariates included the following: age, gender, ethnicity, marital status, poverty-income ratio (PIR), education level, diabetes, cardiovascular disease (CVD), smoking status, physical activity, C-reactive protein(CRP), and body mass index (BMI). Marital status encompasses being married or cohabiting, widowed, divorced, separated, and never married. Educational level is classified as below high school and above high school. CVD is defined as having ever been diagnosed with congestive heart failure, coronary heart disease, angina/angina pectoris, or heart attack. Diabetes is characterized by any of the following conditions: having ever been diagnosed with diabetes or fasting blood glucose levels ≥126 mg/dL. Smoking status encompasses never smoked, former smoker, and current smoker. Moderate activity over the past 30 days was used to assess physical activity. BMI was calculated as weight/(height)^2^ kg/m^2^. PIR is a ratio of family income to the poverty threshold, and we used CRP to represent the inflammatory state.

### 2.5 Statistical analysis

We analyzed the data using EmpowerStats. Categorical variables were presented as survey-weighted percentages (95% CIs), while continuous variables were expressed as survey-weighted means with corresponding 95% confidence intervals (CIs). We present the distribution of baseline data across various groups for the patients enrolled in this study. The two groups distinguished by AR status were assessed using a weighted Student’s t-test for continuous variables or a weighted chi-squared test for categorical variables. The association between alcohol use patterns, heavy drinking, and AR in both genders was examined through a gender-stratified analysis. The linear relationships between average alcohol intake and AR were investigated using weighted multiple linear regression and logistic regression. In each regression analysis, a set of three statistical models was developed in succession. Model I: unadjusted. Model II: adjusted for age, gender, and ethnicity. Model III: adjusted for age, gender, ethnicity, marital status, PIR, education level, diabetes, CVD, smoking status, physical activity, CRP, and BMI. *p*-values below 0.05 were considered statistically significant. *p < 0.05, ** *p* < 0.01, *** *p* < 0.001.

## 3 Results

### 3.1 Baseline characteristics

**Figure 2:**
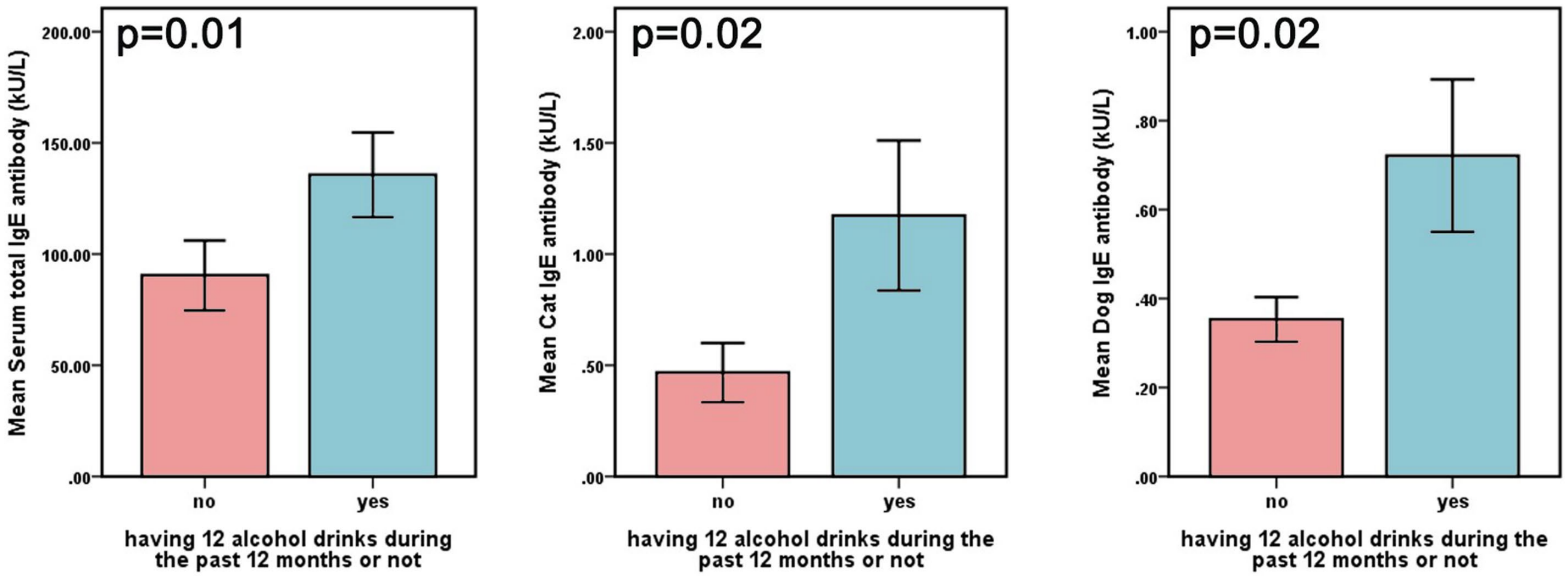
Levels of total IgE and cat/dog dander-specific lgE in different groups based on current alcohol use.

A total of 2,179 participants from the 2005–2006 NHANES cycle were included in the present analysis. Table 1 presents the clinical characteristics of the participants, categorized by the presence of AR. The overall prevalence of current AR among participants was 31.71%. The mean age was 48.14 ± 18.41 years, and 53.42% (*n* = 1,164) were women. Additionally, 65.58% (*n* = 1,429) were married, 73.61% (*n* = 1,604) had completed at least high school education, 53.14% (*n* = 1,158) had never smoked, and 56.49% (*n* = 1,231) were physically active. In addition, the incidence of diabetes and CVD was 11.38 and 7.94%, respectively. We examined 19 allergen-specific IgE levels in individuals with or without alcohol consumption. Our data showed that individuals who currently consumed alcohol exhibited higher levels of cat/dog dander-specific IgE and total IgE compared to those who abstained from drinking in the past 12 months (Figure 2).

**Table 1:** Participants’ baseline characteristics in the study (*N* = 21.79).

Characteristic	Control *N* = 1.488	AR *N* = 691	*p*-value
Age, mean ± SD (year)	49.49 ± 18.78	45.26 ± 17.25	**<0.001**
Gender (*n*)			0.094
Male	675 (45.36%)	340 (49.20%)	
Female	813 (54.64%)	351 (50.80%)	
Ethnicity (*n*)			**<0.001**
Mexican American	337 (22.65%)	100 (14.47%)	
Other Hispanic	47 (3.16%)	24 (3.47%)	
Non-Hispanic White	771 (51.81%)	377 (54.56%)	
Non-Hispanic Black	284 (19.09%)	164 (23.73%)	
Other ethnicity	49 (3.29%)	26 (3.76%)	
Education level (*n*)			**<0.001**
<High School	457 (30.71%)	118 (17.08%)	
≥High School	1,031 (69.29%)	573 (82.92%)	
Marriage			**<0.001**
Never married	171 (11.49%)	129 (18.67%)	
Married	999 (67.14%)	430 (62.23%)	
Widowed	318 (21.37%)	132 (19.10%)	
Diabetes			0.701
No	1,316 (88.44%)	615 (89.00%)	
Yes	172 (11.56%)	76 (11.00%)	
CVD			0.181
No	1,362 (91.53%)	644 (93.20%)	
Yes	126 (8.47%)	47 (6.80%)	
Smoking status			0.096
Never	770 (51.75%)	388 (56.15%)	
Now	309 (20.77%)	141 (20.41%)	
Former	409 (27.49%)	162 (23.44%)	
Physical activity			**<0.001**
no	690 (46.37%)	258 (37.34%)	
yes	798 (53.63%)	433 (62.66%)	
Alcohol consumption pattern			0.09
Never	228 (15.32%)	85 (12.30%)	
Now	1,104 (74.19%)	542 (78.44%)	
Former	156 (10.48%)	64 (9.26%)	
PIR	2.68 ± 1.60	2.91 ± 1.65	**0.002**
CRP (mg/dL)	0.46 ± 0.70	0.46 ± 0.94	0.884
BMI (kg/m^2^)	28.67 ± 6.64	29.14 ± 7.09	0.129

Mean ± SD for continuous variables. The P-value was determined using a weighted Student’s t-test for continuous variables or a weighted chi-square test for categorical variables. Bold values indicate statistical significance of p-value less than 0.05.

### 3.2 Association between alcohol use and AR

It is commonly acknowledged that gender plays a significant biological role in alcohol consumption (18–20). Previous research has also demonstrated the existence of gender differences in alcohol pharmacokinetics, as well as in its effects on neurotransmitter systems and gonadal steroid hormones (21, 22). Therefore, we employed three models to analyze the relationship between alcohol consumption and allergic rhinitis in male and female (Table 2). We observed that, in men, the state of being “Now” (currently drinking) was positively correlated with AR in both the partially and fully adjusted models. However, no differences were observed between “Former” (those with a prior history of drinking but no longer drinking) and “Never” (consuming fewer than 12 alcoholic drinks in a lifetime). We did not find any positive correlation between alcohol consumption patterns and AR in the female group or the overall group. This suggests that current alcohol consumption is associated with a higher incidence of AR in men but not in women, highlighting the influence of gender stratification on these relationships.

**Table 2:** Association between alcohol consumption patterns and the risk of AR.

Alcohol consumption pattern	Model I	Model II	Model III
Male			
Never	1	1	1
Now	1.70 (0.94, 3.09)	1.95^*^ **(1.06, 3.59)**	**2.21** ^*^ **(1.18, 4.15)**
Former	1.22 (0.57, 2.63)	1.50 (0.69, 3.28)	1.79 (0.81, 3.97)
Female			
Never	1	1	1
Now	1.15 (0.84, 1.58)	1.12 (0.80, 1.55)	1.05 (0.74, 1.50)
Former	1.09 (0.70, 1.72)	1.08 (0.68, 1.71)	1.02 (0.64, 1.63)
Total			
Never	1	1	1
Now	1.27 (0.96, 1.67)	1.24 (0.93, 1.65)	1.25 (0.93, 1.69)
Former	1.08 (0.74, 1.59)	1.11 (0.75, 1.65)	1.13 (0.76, 1.69)

Model I: unadjusted.

Model II: adjusted for age, gender, and ethnicity.

Model III: adjusted for age, gender, ethnicity, marriage, PIR, education level, diabetes, CVD, smoking status, physical activity, CRP, and BMI.

^*^p < 0.05. Bold values indicate statistical significance of *p*-value less than 0.05.

Then, we explored whether average alcohol intake and excessive alcohol consumption influence AR. As shown in Tables 3 and 4, after adjusting for all covariates, the *β* values obtained suggested that there is no significant association between an increase or decrease in average alcohol intake and AR promotion. Meanwhile, there was no difference in the development of AR between individuals consuming five or more alcoholic beverages daily and those who do not. These results indicated that while drinking behavior is linked to AR incidence in men, the quantity consumed is not a determining factor.

**Table 3:** Associations between heavy alcohol consumption and the risk of AR.

Heavy alcohol drinking	Model I	Model II	Model III
Male			
No	1.00	1.00	1.00
Yes	0.98 (0.72, 1.34)	1.04 (0.76, 1.43)	1.14 (0.81, 1.59)
Female			
No	1.00	1.00	1.00
Yes	1.48 (0.86, 2.56)	1.40 (0.81, 2.43)	1.80 (0.99, 3.26)
Total			
No	1.00	1.00	1.00
Yes	1.08 (0.83, 1.42)	1.10 (0.84, 1.44)	1.26 (0.94, 1.67)

Model I: unadjusted.

Model II: adjusted for age, gender, and ethnicity.

Model III: adjusted for age, gender, ethnicity, marriage, PIR, education level, diabetes, CVD, smoking status, physical activity, CRP, and BMI.

**Table 4:** Associations between average alcohol intake and the risk of AR.

Average alcohol intake	Model I	Model II	Model III
Male	**0.95** ^*^ **(0.90, 1.00)**	0.95 (0.89, 1.00)	0.95 (0.90, 1.01)
Female	0.99 (0.89, 1.10)	0.96 (0.86, 1.08)	1.01 (0.89, 1.14)
Total	**0.96** ^*^ **(0.91, 1.00)**	**0.95** ^*^ **(0.90, 1.00)**	0.96 (0.91, 1.01)

Model I: unadjusted.

Model II: adjusted for age, gender, and ethnicity.

Model III: adjusted for age, gender, ethnicity, marriage, PIR, education level, diabetes, CVD, smoking status, physical activity, CRP, and BMI.

^*^p < 0.05. Bold values indicate statistical significance of *p*-value less than 0.05.

## 4 Discussion

In the NHANES cross-sectional study involving 2,179 adults from the US, we found that current alcohol consumption was linked to a higher risk of AR in men but not in women.

The morbidity and mortality associated with allergic illnesses are both on the rise, and dietary and lifestyle factors may contribute to this increase (23, 24). For example, adults who currently smoke may experience more severe respiratory and rhinitis symptoms (25). Clinical evidence suggests that children with AR who are exposed to passive smoke and exhibit elevated serum cotinine levels may develop more severe nasal obstruction and microbial dysbiosis (26). Meanwhile, observational analyses have revealed that milk consumption has a protective effect against hay fever and asthma (27). Certain nutrients, including retinol, vitamin A, cryptoxanthin, copper, and zinc, may also have positive effects on reducing allergic symptoms (28, 29). Furthermore, numerous studies have examined the effects of alcohol on AR and other allergic diseases (6, 7, 9–11, 30), but no unanimous conclusion has been reached.

Alcohol consumption, even in moderation, can influence the immune response and have negative effects on health (30, 31). After alcohol consumption, it enters the bloodstream from the stomach and the small intestine, diffuses various organs, is then metabolized into acetaldehyde and acetate (32). Binge drinking and chronic drinking alter the ability of macrophages to detect pathogens and affect the production of proinflammatory cytokines by dendritic cells (DCs), such as IL-1β, TNF-*α*, IL-6, and IL-12 (33–35). Furthermore, chronic moderate drinking promotes the activation and proliferation of T and B cells, while chronic heavy drinking is linked to the depletion and apoptosis of T and B cells, as well as an increase in immunoglobulins (36). The positive effects of alcohol on IgE and IL-13, while IL-4 levels remain unchanged, are primarily derived from observational studies and animal experiments (9, 10, 37–39). Given that alcohol can alter the epigenetic landscape of genes—affecting gene transcription through mechanisms such as miRNAs, DNA methylation, and histone modifications (31)—similar processes may contribute to these effects. However, further research is necessary to explore and clarify the underlying mechanisms.

Further research is required to determine the mechanisms through which alcohol consumption impacts AR. Additionally, the gender differences in the correlation between alcohol consumption patterns and AR are intriguing. Studies have shown that respiratory allergies, particularly asthma, are more common in men during childhood, while they tend to become more prevalent in female individuals from adolescence or after menarche into adulthood, mainly due to the effect of female hormones, particularly the modulation of inflammatory responses by estrogens (40, 41).

Excessive alcohol consumption lowers testosterone levels by increasing the activity of aromatase, which converts testosterone into estradiol (21). Androgens inhibit Th2 differentiation and Th2 memory formation (42), while androgen receptor signaling reduces allergic airway inflammation (43). Conversely, estrogen and progesterone promote type 2 responses and suppress type 1 responses (40). It is still unknown whether sex hormones act as an intermediate factor in the relationship between alcohol consumption patterns and AR. Therefore, clinical cohort studies and animal experiments are needed.

This current study has several limitations. First, because NHANES is a cross-sectional database without longitudinal follow-up, it is impossible to determine the mechanism of the observed association or the direction of causation. Second, the assessment of AR diagnosis was limited by the lack of clinical symptom observation and the pattern of alcohol consumption—the exposure variable was determined through interviews and recall, which may have introduced misclassification bias. Third, potential confounding factors such as medication use, dietary habits, and residential characteristics were not collected in the NHANES data bank. Finally, further research with a larger sample size is necessary.

In summary, we discovered that current alcohol consumption is positively associated with a higher prevalence of AR in men. This insight may contribute to a better understanding of AR and help improve the quality of life for AR patients.

## Data Availability

Publicly available datasets were analyzed in this study. This data can be found at: https://www.cdc.gov/nchs/nhanes/.
